# Outcomes of Pregnancies with Absent or Hypoplastic Fetal Nasal Bone: A Retrospective Analysis of Prenatal Findings and Perinatal Outcomes

**DOI:** 10.3390/life15081215

**Published:** 2025-08-01

**Authors:** Eva Karner, Lara Krepler, Petra Pateisky, Agnes Grill, Paul Dremsek, Guelen Yerlikaya-Schatten, Stephanie Springer

**Affiliations:** 1 Department of Obstetrics and Gynecology, Division of Obstetrics and Feto-Maternal Medicine, Medical University of Vienna, Spitalgasse 23, 1090 Vienna, Austria; eva.karner@meduniwien.ac.at (E.K.); lara.krepler@meduniwien.ac.at (L.K.); petra.pateisky@meduniwien.ac.at (P.P.); stephanie.springer@meduniwien.ac.at (S.S.); 2 Department of Pediatrics and Adolescent Medicine, Division of Neonatology, Intensive Care Medicine and Neuropediatrics, Medical University of Vienna, Spitalgasse 23, 1090 Vienna, Austria; agnes.grill@meduniwien.ac.at; 3 Institute of Medical Genetics, Medical University of Vienna, Waehringer Strasse 10, 1090 Vienna, Austria; paul.dremsek@meduniwien.ac.at

**Keywords:** hypoplastic fetal nasal bone, absent fetal nasal bone, chromosomal anomalies, aneuploidy, first-trimester screening, nasal bone dysplasia, nasal bone anomaly, trisomy 21

## Abstract

Hypoplastic or absent fetal nasal bone (NB) is a significant soft marker in the risk assessment for aneuploidies. This study aimed to evaluate prenatal findings and perinatal outcomes in fetuses with absent or hypoplastic NB managed at our center. This retrospective analysis was conducted at the Department of Obstetrics at the Medical University of Vienna and including all cases with an absent or hypoplastic fetal NB between 2004 and 2022. Clinical data were extracted and analyzed using descriptive statistics. A total of 149 cases were included. Of these, 51% had chromosomal abnormalities, with trisomy 21 present in 30.9%. Malformations were identified in 55% of cases, most commonly congenital heart defects (34.9%) and facial dysmorphism (28.9%). Eighteen fetuses (12.1%) had structural anomalies without genetic disorders. In 32.9% (n = 49), the NB anomaly was isolated. Our findings show that only half of the cases had chromosomal abnormalities, and over half of the pregnancies resulted in live births with generally favorable perinatal outcomes. However, the presence of additional ultrasound abnormalities significantly increased the risk of adverse outcomes. Therefore, detection of a fetal NB anomaly should prompt comprehensive ultrasound evaluation and genetic testing.

## 1. Introduction

The fetal nasal bone (NB) is an additional ultrasound marker for chromosomal anomalies and can typically be visualized during first-trimester screening between 11 + 0 and 14 + 0 weeks of gestation. The examination of the fetal NB is part of the combined first-trimester screening, which additionally includes maternal age, gestational age, maternal history of pregnancies with chromosomal anomalies, further ultrasound parameters such as nuchal translucency (NT) thickness, and serum biomarkers including free beta-human chorionic gonadotropin (β-hCG) and pregnancy-associated plasma protein A (PAPP-A) [1,2]. The visualization of the fetal NB is performed by using the midsagittal plane and should indicate the tip of the nose and the anterior palate with its rectangular shape. The NB appears more echogenic than the skin above [3]. An absent or hypoplastic NB is observed in approximately 36–70% of fetuses with diagnosed trisomy 21 [3,4,5]. However, an absent or hypoplastic NB may also be associated with other chromosomal anomalies [6].

Cases with an isolated absent or hypoplastic NB, without other structural anomalies, are less likely to be associated with aneuploidy [7,8,9]. According to the literature, 0.5% to 2.8% of euploid fetuses are reported to have an absent NB [6,10]. A short or hypoplastic NB may represent a normal variant or may be more common in certain ethnic groups. Patients with Asian or African Caribbean ancestry are more likely to have a fetus with a short fetal NB [6,11]. Risk stratification for aneuploidy should therefore take ethnic origin into account [11,12].

An algorithm to calculate the individual risk for common aneuploidies is recommended by the Fetal Medicine Foundation (FMF) [13]. Previously, detection rates of over 90% for trisomy 21, trisomy 18, trisomy 13, and Turner syndrome have been reported [14,15]. Aneuploidies are associated with increased rates of spontaneous intrauterine fetal demise (IUFD), as well as higher neonatal mortality and morbidity [16]. Furthermore, children with an aneuploidy who survive the neonatal period may require lifelong medical care. Families with an increased risk for aneuploidy in the current pregnancy must be counseled regarding further prenatal testing, options for the ongoing pregnancy, and potential pre- and postnatal treatments. Possible benefits, risks, and limitations of all available options should be thoroughly discussed [2,17]. Additional examinations of the fetal organs should be conducted to guarantee a proper interdisciplinary perinatal care of the newborn. Therefore, these patients should be referred to a tertiary care center [18].

Our study presents data from a tertiary care center on pregnancy outcomes and genetic analyses of cases with prenatally diagnosed absent or hypoplastic fetal NB. The aim of our study is to contribute to the existing knowledge on this topic and to support patient counseling, particularly in cases of absent or hypoplastic fetal NB with a normal karyotype or an isolated NB.

## 2. Material and Methods

This single-center, retrospective, observational study was performed at the Department of Obstetrics and Gynecology, Division of Obstetrics and Feto-maternal Medicine, a tertiary care center for fetal medicine at the Medical University of Vienna, Austria. The study was authorized by the institutional ethics committee (1221/2023) and was performed according to the standards of the Helsinki Declaration. Pseudonymized data from medical records were used; thus, patient participation was not necessary. Nevertheless, following an internal hospital standard operating procedure, written declaration of consent was obtained at registration.

Medical data from all patients with an absent or hypoplastic fetal NB between 2004 and 2022 were extracted from our records using Viewpoint^®^ software, Version 5.6.28.56 (GE Healthcare, Wessling, Germany). Sonographic assessment of the fetal nasal bone was conducted according to the ISUOG guidelines on first trimester screening or second trimester screening by experienced sonographers certificated by the FMF [2]. The fetal nasal bone was diagnosed as absent if it could not be demonstrated or hypoplastic if it was measured with a length under 2.5th percentile regarding the publication by Cicero et al. [3]. Patients who were lost to follow-up and multiple pregnancies were excluded. Also second-trimester screenings were performed by certified sonographers in accordance with ISUOG guidelines [18]. If the measurement in the second trimester showed a normal fetal nasal bone, the patient was excluded from the analysis. Additional soft markers in the second trimester included nuchal edema, single umbilical artery, hyperechoic or dilated intestine, choroid plexus cyst, as well as polyhydramnios and oligohydramnios. Prenatal cytogenetic analysis was offered to all patients, tailored to the individual clinical scenario. This analysis could include karyotyping, fluorescence in situ hybridization (FISH), chromosomal microarray analysis (CMA), or whole exome sequencing (WES), depending on the clinical indication, gestational age, and the year in which the patient was examined. The choice of test was made in consultation with the patient and based on current diagnostic standards at the time of evaluation. Patients’ parameters on obstetric and neonatal outcome, such as termination of pregnancy, intrauterine fetal demise, gestational age at delivery, birth weight, and perinatal and neonatal mortality, were extracted and analyzed. Gestational age was based on the first dating scan recorded or on first-trimester screening following the ISUOG practice guidelines [2]. Fetal growth restriction was diagnosed in fetuses with an estimated fetal birth weight under the 3rd percentile [19]. Preterm birth was defined as birth before gestational age of 37 weeks. Stillbirth was considered as fetal death after gestational week 20 + 0; early neonatal mortality was defined as death within the first 7 days of life [20,21]. An isolated NB anomaly was defined as a NB abnormality in the absence of any other ultrasound findings (e.g., malformation, soft marker, or nuchal translucency > 3.5 mm).

### Statistical Analysis

Statistical analysis was performed using SPSS version 29 for MAC (IBM SPSS Inc., Armonk, New York, NY, USA) and reported as mean (±standard deviation) for normally distributed continuous variables and median (interquartile range) for non-normally distributed continuous variables. A Kolmogorov–Smirnov test was used to identify non-normally distributed continuous variables. In case of continuous variables, the two groups were compared using the Student’s *t*-test or Mann–Whitney U-test, as appropriate. Categorical variables were analyzed with the chi-square test or Fisher’s exact test. Differences were considered statistically significant if *p*-values ≤ 0.05.

## 3. Results

Our database provided 196 patients who met our inclusion criteria. Three patients could not be analyzed due to missing data. Twenty-seven patients had an initially suspicious NB finding in the first-trimester screening that could not be confirmed in a subsequent scan and therefore were excluded from our analysis. Seventeen patients were excluded because they had multiple pregnancies. Therefore, 149 patients were included in the final analysis. Maternal characteristics are presented in Table 1. Overall, our patients were generally young and healthy patients. Seven patients (4.7%) conceived through assisted reproductive technology (ART). Furthermore, we observed five cases (3.4%) of consanguinity in our cohort. As the study was conducted at a tertiary referral center, patients were mainly referred to our department due to an abnormal combined test in the outpatient setting (33/149, 22.15%). Six (4%) patients were referred due to an abnormal fetal NB, nine (6%) due to an abnormal nuchal translucency and thirty-two (21.4%) for suspected fetal anomaly. In five (3.3%) cases, chorionic sampling was already performed in the outpatient setting; in contrast, four (2.68%) patients were referred to chorionic sampling or amniocentesis. However, we provide first- and second-trimester screenings for all women who register for delivery at our hospital. Therefore, 49 (32.8%) patients with absent or hypoplastic fetal NB were detected through regular screening.

In 104 cases (69.8%), the fetal NB was absent; in 38 cases (25.5%), it was described as hypoplastic; and in 7, cases (4.7%), ultrasound findings of the NB were unclear. The severity of the NB abnormality was not significantly associated with differences in live birth rates, survival, termination of pregnancy (TOP), or genetic aberrations. However, fetuses with a hypoplastic or unclear NB had significantly more additional malformations than those with an absent NB (*p* = 0.027).

Regarding prenatal diagnostics, first-trimester screening was classified as abnormal in 117 cases (78.5%), with a calculated high risk for chromosomal anomalies. A nuchal translucency (NT) > 3.5 mm was observed in 41 fetuses (27.5%). Pregnancies with a nuchal translucency (NT) > 3.5 mm were significantly associated with the occurrence of additional malformations (*p* = 0.023); soft markers (*p* < 0.001); fetal hydrops (*p* < 0.001); chromosomal aberrations (*p* < 0.001), especially trisomy 21 (*p* = 0.014); abnormal second trimester scans (*p* = 0.044); and fetal death (*p* < 0.001). Overall, 128 families (85.9%) opted for genetic testing, including both non-invasive and invasive methods. A total of 106 patients (71.1%) underwent invasive diagnostics, including 35 patients (22.5%) with amniocentesis, 10 patients (6.7%) with placental puncture, and 65 patients (43.6%) with chorionic villus sampling. In some patients, multiple invasive procedures had to be performed. Non-invasive genetic testing was conducted using fetal cell-free DNA and showed a positive result in 34 patients (22.8%). In 21 patients (14.4%), genetic testing was not conducted. Of all patients, 76 patients (51%) showed genetic aberrations. Forty-six fetuses (30.9%) were diagnosed with trisomy 21, nine fetuses (6%) with trisomy 13, and seven fetuses (4.7%) with trisomy 18. Other chromosomal aberrations, including Turner syndrome, Fraser syndrome, a microduplication in 5q21.1, a microdeletion in 17q22, a deletion on chromosome 2, as well as trisomies of chromosomes 7 and 9 and mosaicisms, were found in 14 patients (9.4%). Women carrying fetuses with genetic anomalies were significantly older (*p* < 0.001). The median age of women with fetal chromosomal abnormalities was 37 years (33–40) compared to a median age of 30 years (25–37) in those without chromosomal aberrations. Pregnancies with an NT > 3.5 mm (*p* < 0.001), an abnormal ductus venosus (*p* = 0.017), or soft markers (*p* < 0.001) had a significantly higher rate of chromosomal abnormalities. Moreover, fetuses without genetic abnormalities had a significantly lower incidence of additional malformations (34.6% vs. 72.4%, *p* < 0.001). In 55 patients (36.9%), the second-trimester screening was abnormal, whereas in 61 cases (40.9%), second-trimester screening results were not available. Patients with an abnormal second-trimester screening had significantly more common chromosomal aberrations (*p* < 0.001) and additional malformations (*p* < 0.001). Moreover, fetuses with a hypoplastic or unclear NB had a significantly higher rate of abnormal second-trimester screenings compared to fetuses with an absent NB (*p* < 0.001). A significantly higher rate of abnormal second-trimester screenings was observed in pregnancies associated with TOP (*p* < 0.001), trisomy 21 (*p* < 0.001), preterm birth (*p* = 0.024), neonatal intensive care unit (NICU) transfer (*p* = 0.019), or fetal growth restriction (FGR) (*p* = 0.002). Eighty-two fetuses (55%) had additional malformations, most commonly involving the heart (52 fetuses, 34.9%), followed by facial dysmorphism (43 fetuses, 28.9%). Pregnancies with additional malformations showed a significantly higher occurrence of ultrasound soft marker (*p* < 0.001), genetic aberrations (*p* < 0.001), and termination of pregnancy (*p* < 0.001). Fetuses with fetal growth restriction (*p* = 0.038) and those who did not survive (*p* < 0.001) had a significantly higher incidence of additional malformations. Eighteen fetuses (12.1%) were diagnosed with malformations in the absence of chromosomal aberrations. Additional soft markers were found in 56 patients (37.6%). Fetal growth restriction (FGR) was prenatally diagnosed in only 13 pregnancies (8.7%), while fetal hydrops occurred in 34 pregnancies (22.8%). Women carrying fetuses with additional malformations were significantly older (*p* = 0.022). The median age in this group was 35 years (29–39) compared to 32 years (26–37) in women without additional malformations. Malformations and other prenatal findings are listed in Table 2.

An isolated NB anomaly was found in 49 cases (32.9%). Nine (18.4%) of these were confirmed to have genetic aberrations, including six cases of Down syndrome and three cases with other genetic anomalies. Pregnancies with an isolated hypoplastic or absent fetal NB had significantly lower rates of TOP and genetic aberrations, particularly trisomy 21 (all *p* < 0.001). In addition, these fetuses had significantly higher live birth and survival rates (both *p* < 0.001). Of all 49 cases, 39 (79.6%) had genetic testing, of which 9 (23.0%) showed chromosomal aberration. The rest (30/49) had no aneuploidy; they were either terminated or aborted. All of those isolated NB anomalies without genetic aberrations were live births; only three were delivered premature, and no neonatal mortality was detected.

Of all cases, 65 families (43.6%) opted for TOP, while 9 pregnancies ended in spontaneous abortion (6%), and 3 resulted in stillbirth (2%). One of the three stillbirths showed no chromosomal abnormality, while in another case, genetic testing was declined by the family. One of the three stillbirths had an isolated NB anomaly. Pregnancies with TOP showed a significantly higher incidence of additional malformations (*p* < 0.001) and genetic anomalies (*p* = 0.003). Seventy-two fetuses (48.3%) diagnosed with an absent or hypoplastic NB were born alive. Fetuses with an isolated NB anomaly or without genetic anomalies had significantly higher survival and live birth rates and significantly lower rates of TOP (all *p* < 0.001). Pregnancy outcomes are presented in Table 3.

Of all live births, 16 pregnancies (22.2%) resulted in preterm delivery due to preterm premature rupture of membranes (PPROM), fetal growth restriction, or maternal indications such as preeclampsia. Three newborns died within the neonatal period. Neonatal death occurred in one newborn with trisomy 18 and one with trisomy 13. The third was diagnosed with multiple malformations, including a complex heart defect. All three died within the first 24 h. In each case, the parents declined termination of pregnancy and opted for perinatal comfort care. Twenty newborns (27%) required transfer to the neonatal intensive care unit (NICU) and survived until discharge. Preterm newborns (*p* = 0.011) and those with malformations (*p* < 0.001) or FGR (*p* = 0.010) were significantly more often transferred to the NICU. Newborns with an isolated NB anomaly were significantly less likely to be transferred to the NICU (*p* < 0.001) and overall showed good birth characteristics regarding birthweight, pH of the umbilical cord, and APGAR. Characteristics of live births are shown in Table 4.

## 4. Discussion

This retrospective study, conducted at a tertiary referral center for fetal medicine, analyzes the perinatal outcomes of 149 fetuses diagnosed with an absent or hypoplastic NB.

Our cohort showed chromosomal aberrations in 51% of cases. Only one-third (30.9%) of all cases were diagnosed with Down syndrome. A total of 9.4% were diagnosed with rare chromosomal aberrations. In the literature, however, an absent or hypoplastic fetal NB has been repeatedly described as a marker for aneuploidy, particularly trisomy 21 [3,9,10,15,22,23,24]. Cicero et al. reported an absent NB in up to 73% of fetuses with trisomy 21 and in 0.5% of chromosomally normal fetuses [3].

We also examined outcomes in patients without chromosomal anomalies and observed spontaneous abortion in 3.8% and stillbirth and neonatal death each in 1.9%. This cohort had a significantly lower incidence of additional malformations. Notably, all fetuses with adverse outcomes had additional malformations. Furthermore, children without genetic abnormalities exhibited higher rates of live birth and survival. Pregnancies in which the fetus presented with an isolated NB anomaly were also associated with higher live birth and survival rates in our cohort. Most live births had favorable perinatal outcomes. Only three newborns died within the first 24 h of life; all were diagnosed with genetic aberrations and/or multiple malformations. Dukhovny et al. investigated perinatal outcomes in 28 fetuses with an absent NB and similarly found that adverse outcomes occurred only in cases with additional abnormal sonographic findings [23].

Overall, our analysis highlights that fetuses with additional malformations, abnormal sonographic findings, and soft markers are at higher risk for adverse perinatal outcomes compared to those with an isolated NB abnormality. More than half of the fetuses in our cohort (55%) were diagnosed with additional malformations. Congenital heart defects (CHDs) were the most commonly identified organ anomalies in our cohort, which aligns with the existing literature, as CHDs are strongly associated with chromosomal abnormalities, particularly trisomy 21 [25,26]. Facial dysmorphism was the second most common malformation observed in our cohort. A previous study reported that NB anomalies can be associated with craniofacial anomalies [6]. The presence of additional malformations was associated with an increased risk of genetic aberrations and a higher likelihood of pregnancy termination. A significantly higher incidence of additional malformations was observed in fetuses with fetal growth restriction and in those who did not survive. Our findings are consistent with previous studies reporting that fetuses with additional sonographic abnormalities are at increased risk of stillbirth as well as higher perinatal morbidity and mortality [6,9,23,27].

The meta-analysis by Agathokleous et al. concluded that the presence of multiple soft markers in combination with fetal NB dysplasia significantly increases the risk of trisomy 21 [27]. We also found that the presence of soft markers was significantly associated with a higher rate of chromosomal abnormalities. In our cohort, a nuchal translucency measurement greater than 3.5 mm showed significant association not only with genetic aberrations but also with adverse perinatal outcomes. Fantasia et al. concluded that a thickened nuchal translucency is a stronger predictor of genetic disorders than an absent or hypoplastic NB [28].

Forty-nine cases of fetal NB dysplasia in our cohort showed no malformations or additional soft markers, including increased nuchal translucency, and were therefore classified as isolated NB anomalies. Six fetuses (12.2%) with isolated NB dysplasia were diagnosed with Down syndrome. Lostchuck et al. analyzed a comparable number of cases with isolated hypoplastic NB and similarly reported a 10% incidence of trisomy 21 [29]. Shi et al. described a 9% incidence of chromosomal aneuploidy in fetuses with isolated NB dysplasia, with Down syndrome being the most frequent abnormality [30]. In a study of 55 cases with isolated NB hypoplasia or absence, Zhang et al. found an abnormal karyotype in 7.27% and additional pathogenic findings via whole exome sequencing (WES) in 16.67%. They therefore recommended further genetic testing in cases of isolated NB abnormalities, even in the absence of other malformations or soft markers [31]. Based on these findings, the Society for Maternal-Fetal Medicine recommends counseling regarding the estimated risk of Down syndrome and offering non-invasive prenatal testing (NIPT) using cell-free DNA (cfDNA), if not already performed. If cfDNA testing is not feasible, amniocentesis may be considered. However, if cfDNA results are negative for aneuploidy in cases of isolated fetal NB dysplasia, no further evaluation is deemed necessary [32].

However, they do not differentiate between an absent and a hypoplastic fetal NB in their recommendation. Shi et al. analyzed data from 229 fetuses with an isolated absent or hypoplastic NB and reported a higher detection rate of chromosomal aberrations in fetuses with an absent NB compared to those with a hypoplastic NB [30]. The study by Zhang et al. yielded controversial results; however, it included only second-trimester detections of abnormal fetal NB [31]. Apart from an increased risk of additional malformations in fetuses with hypoplastic or abnormal NB, the type of NB anomaly had no impact on fetal outcome in our cohort. No significant difference in the rate of genetic aberrations was observed, although there was a slight tendency toward a higher rate in fetuses with a hypoplastic NB (hypoplastic 75.8% vs. absent 54.5% vs. abnormal 42.9%; *p* = 0.07).

Variations in NB length based on a patient’s demographic background have been well documented. Several studies from different countries suggest that reference values for NB length should be adjusted according to the patient’s ethnicity [12,33,34]. Due to missing data on patient origin, we were unable to evaluate ethnic variations in NB length, which represents a limitation of our study.

In addition, advanced maternal age was significantly associated with chromosomal aberrations, a correlation that has been well established in previous studies [35].

According to the current literature, consanguinity is considered a risk factor for abnormal karyotypes, organ malformations, and other abnormal ultrasound findings [36]. However, in our cohort, consanguinity was not overrepresented and showed no association with genetic abnormalities or sonographic anomalies. Thus, based on our findings, consanguinity does not appear to have a causative association with a hypoplastic or absent fetal NB.

Regarding the measurement of fetal NB, it has to be discussed that the accuracy of the measurement of the fetal NB depends on observer variability underlying the nature of sonographic evaluations. There have been studies on interobserver variability showing conflicting results regarding the reproducibility of the measurement [37,38,39]. As this is a retrospective study, interobserver variability could not be analyzed.

## 5. Strengths and Limitations

Our study presents perinatal outcomes in fetuses with and without chromosomal anomalies, providing valuable insights into euploid cases with absent or hypoplastic NB. It highlights outcomes in isolated NB dysplasia and includes rare cases of chromosomal aberrations. However, the retrospective design may introduce bias. A key limitation is the lack of information regarding the patients’ ethnic backgrounds, which is relevant given known ethnic variations in fetal NB length. Additionally, the actual NB measurements were not documented in cases classified as hypoplastic. Data on non-invasive prenatal testing (NIPT) were incomplete, as many tests were performed externally; we were only able to assess abnormal NIPT results. While most live births in our cohort had favorable perinatal outcomes, our analysis is limited to birth characteristics and early neonatal outcomes and does not include data on long-term neonatal development.

## 6. Conclusions

**Figure 1:**
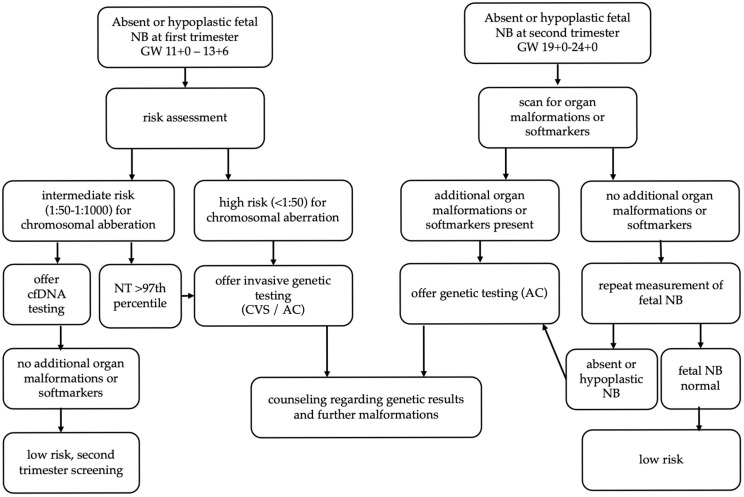
Flow chart recommendation for the evaluation of fetal NB anomaly based on our findings. fetal NB, nasal bone; cfDNA, cell free DNA; CVS, chorionic villous sampling; AC, amniocentesis.

This study presents prenatal findings and perinatal outcomes in cases with absent or hypoplastic fetal NB. Chromosomal anomalies and additional malformations were identified in only half of the cases, with the majority of newborns demonstrating favorable perinatal outcomes. The risk of chromosomal abnormalities—and consequently adverse neonatal outcomes—increased significantly when additional ultrasound findings such as structural malformations or soft markers were present. If an NB anomaly is detected during first-trimester screening, it should be included in the overall risk assessment, and counseling for further diagnostics should be based on the calculated risk. A thorough evaluation for additional anomalies is essential. If a NB anomaly is detected during the second-trimester screening, a genetic evaluation—either via cfDNA testing or amniocentesis—should be offered based on prior findings and any additional malformations. Figure 1 shows a flow chart for the evaluation of fetal NB anomaly based on our findings.

## Figures and Tables

**Table 1:** Maternal characteristics.

Variable	N = 149
Maternal age ^1^	34 (27–39)
Body mass index ^1^	23.5 (21–26)
Smoking ^2^	18 (12.1)
Body mass index > 24.9 kg/m^2^	49 (32.9)
Assisted reproductive techniques ^2^	7 (4.7)
Consanguinity	5 (3.4)
Nulliparity ^2^	43 (28.9)
Gestational diabetes ^2^	13 (8.7)
Hypertensive pregnancy disorders ^2^ *	8 (5.4)
Obstetric complications ^2^ **	13 (8.7)

^1^ Median (interquartile range, IQR), ^2^ number (percentage); * pregnancy-induced hypertension, preeclampsia, and HELLP syndrome; ** preterm rupture of membranes, cervical insufficiency, o preterm contractions.

**Table 2:** Prenatal diagnoses.

Variable	All Cases N = 149	No Chromosomal Aberrations N = 52	Isolated Nasal Bone Anomaly N = 49
Absent nasal bone	104 (69.8)	40 (76.9)	38 (77.5)
Hypoplastic nasal bone	38 (25.5)	8 (15.4)	11 (22.4)
Abnormal nasal bone	7 (4.4)	4 (7.7)	0 (0)
Nuchal translucency > 3.5 mm	41 (27.5)	6 (11.5)	-
Tricuspid regurgitation	9 (6)	3 (5.8)	-
Abnormal ductus venosus	28 (18.8)	10 (19.2)	-
Malformations *	82 (55)	18 (34.6)	-
Heart defects	52 (34.9)	10 (19.2)	-
Facial dysmorphism	43 (28.9)	11 (21.2)	-
Brain malformations	29 (19.5)	5 (9.6)	-
Intestinal malformations	26 (17.4)	7 (13.5)	-
Urogenital malformations	19 (12.8)	5 (9.6)	-
Extremities anomalies	15 (10.1)	4 (7.7)	-
Isolated nasal bone anomaly	49 (32.9)	30 (57.7)	-
Soft marker	56 (37.6)	9 (17.3)	-
Fetal growth restriction	13 (8.7)	4 (7.7)	-
Fetal hydrops	34 (22.8)	2 (3.9)	-
Genetic testing conducted **	128 (85.9)	52 (100)	39 (79.6)
Invasive genetic testing conducted	106 (71.6)	35 (67.3)	24 (48.98)
Amniocentesis	35 (23.5)	14 (26.9)	8 (16.3)
Chorionic villous sampling	65 (43.1)	19 (36.5)	18 (36.7)
Placental puncture	10 (6.7)	5 (9.6)	0 (0)
Non-invasive genetic testing positive	34 (22.8)	18 (34.6)	17 (34.7)
Chromosomal aberration	76 (51)	-	9 (18.4)
Trisomy 21	46 (30.9)	-	6 (12.2)
Trisomy 18	7 (4.7)	-	0 (0)
Trisomy 13	9 (6)	-	0 (0)
Other chromosomal aberrations ***	14 (9.4)	-	3 (6.1)

All parameters described as numbers (percentage); * patients with malformations; categories of malformations describe the number of each category, and multiple categories can be found in one fetus, ** including invasive and non-invasive genetic testing, *** Turner syndrome, Fraser syndrome, microduplication in 5q21.1, microdeletion in 17q22, deletion in chromosome 2 as well as trisomy of chromosomes 7 and 9, and Mosaics.

**Table 3:** Pregnancy outcomes.

Variable	All Cases N = 149	No Chromosomal Aberrations N = 52	Isolated Nasal Bone Anomaly N = 49
TOP	65 (43.6)	6 (11.5)	7 (14.3)
Abortion until 20 weeks GA	9 (6)	2 (3.8)	1 (2)
Stillbirth	3 (2)	1 (1.92)	1 (2)
Live births	72 (48.3)	43 (82.7)	40 (81.6)
Neonatal death	3(2)	1 (1.92)	0 (0)

All parameters described as numbers (percentage). TOP, termination of pregnancy; GA, gestational age.

**Table 4:** Birth characteristics of live births.

Variable	N = 72
GA at birth ^1^	38.57 (37.29–40.00)
Vaginal birth ^2^	34 (47.2)
Preterm birth ^2^	16 (22.2)
Male sex ^2^	29 (40.3)
Birth weight in grams ^1^	3000 (2670–3590)
Birth weight percentile ^1^	34 (9–70)
Birth length in centimeters	50 (48.5–52.3)
APGAR 1 ^1^	9 (8–9)
APGAR 5 ^1^	10 (9–10)
APGAR 10 ^1^	10 (9–10)
pH ^1^	7.27 (7.19–7.32)
Transfer to NICU ^2^ *	20 (27.8)

^1^ Median (IQR); ^2^ number (percentage). * NICU, neonatal intensive care unit.

## Data Availability

The data presented in this study are not publicly due to privacy retrictions, but available on request from the corresponding author.
